# An Introduction to Statistics – Data Types, Distributions and Summarizing Data

**DOI:** 10.5005/jp-journals-10071-23198

**Published:** 2019-06

**Authors:** Priya Ranganathan, Nithya J Gogtay

**Affiliations:** 1 Department of Anesthesiology, Tata Memorial Hospital, Homi Bhabha National Institute, Mumbai, Maharashtra, India; 2 Department of Clinical Pharmacology, King Edward Memorial Hospital, Mumbai, Maharashtra, India

**Keywords:** Biostatistics, Data analysis, Statistics and numerical data

## Abstract

The purpose of research is to gather data, which can then be used to inform decision-making. Data can be of various types and an understanding of this is crucial for its proper analysis and interpretation. In this article, we look at various types and distributions of data, and methods to summarize this data.

**How to cite this article:**

Ranganathan P, Gogtay NJ, An Introduction to Statistics – Data Types, Distributions and Summarizing Data. Indian J Crit Care Med 2019;23(Suppl 2):S169–S170.

## INTRODUCTION

In the first article of this series, we look at types of data and the methods used to describe or summarize data. Data is defined as *‘factual information (such as measurements or statistics) used as a basis for reasoning, discussion, or calculation’.*^1^ As statistics begins with data collection, understanding data is important, as it will help apply the right statistical tests, make the appropriate assumptions and draw meaningful and robust conclusions.

### Classification of Data

At the highest level, data can be broadly classified as **qualitative data** (also known as **categorical data**) or **quantitative data** (also known as **numerical data**).

*Qualitative or categorical data* answers the question ‘What type’ or ‘Which category’. Gender (male/female), marital status (married/divorced/widowed/single), severity of pain on visual analog scale (mild/moderate/severe) are examples of qualitative or categorical data. Within categorical data, there are subtypes. Nominal data is data that does not follow any particular natural order, e.g. marital status (married / divorced/ widowed/ single). Here, there is no sequence between the various categories. In contrast, severity of pain can be ranked in ascending order as none/mild/moderate/severe/unbearable. This is known as *ordinal data*. Categorical data can also be classified depending on the number of categories. If only two categories are present e.g., dead/alive, married/unmarried – then the data is *binary or dichotomous*. If more than two categories are present e.g., married/divorced/widowed/single then this is *non-binary data*. In summary, qualitative data deals with characteristics, traits or descriptors or judgements.

*Note:* While collecting data, it is useful to collect it in as many categories as required so that information is not missed out. Subsequently, if needed, the researcher can rearrange the categories and collapse them into binary data or two categories for ease of analysis and interpretation.

*Quantitative or numerical data* is data that can be counted and answers the question ‘How many’ or ‘How much”. For example, age, height, weight, blood pressure, number of children, number of hospital visits. Numerical data can be further classified into discrete data and continuous data. *Discrete data* is data that can only be counted in whole numbers. e.g., number of hospital visits, Glasgow Coma Score, Richmond Agitation Sedation Scale, modified Rankin scale. This type of data cannot be represented in decimals – so Glasgow Coma Scale score can be 7 or 8 not 7.5. *Continuous data* is data that theoretically has no gap between data points can be counted in decimals (depending on the precision of the measuring instrument) e.g., blood glucose, hemoglobin, serum lipids. In summary, quantitative data deals with objective measurements.

*Note:* The same outcome can be measured as either numerical or categorical data depending on the measuring tool. For example, pain measured on a visual analog scale is categorical data (none/mild/moderate/severe/unbearable). The same outcome can be measured on a numerical rating scale of 0–10 where 0 represents no pain and 10 represents unbearable pain. Thus, the purpose of data is to finally answer the research question and the classification of data is not sacrosanct.

### Distribution of Data

The distribution of data obtained from a sample is crucial to understand how to analyze it. There are various probability distributions for different types of data. Of these, one particularly important type used in everyday research is the distribution of continuous numerical data. This data may either be normally distributed or skewed.

**Fig. 1:**
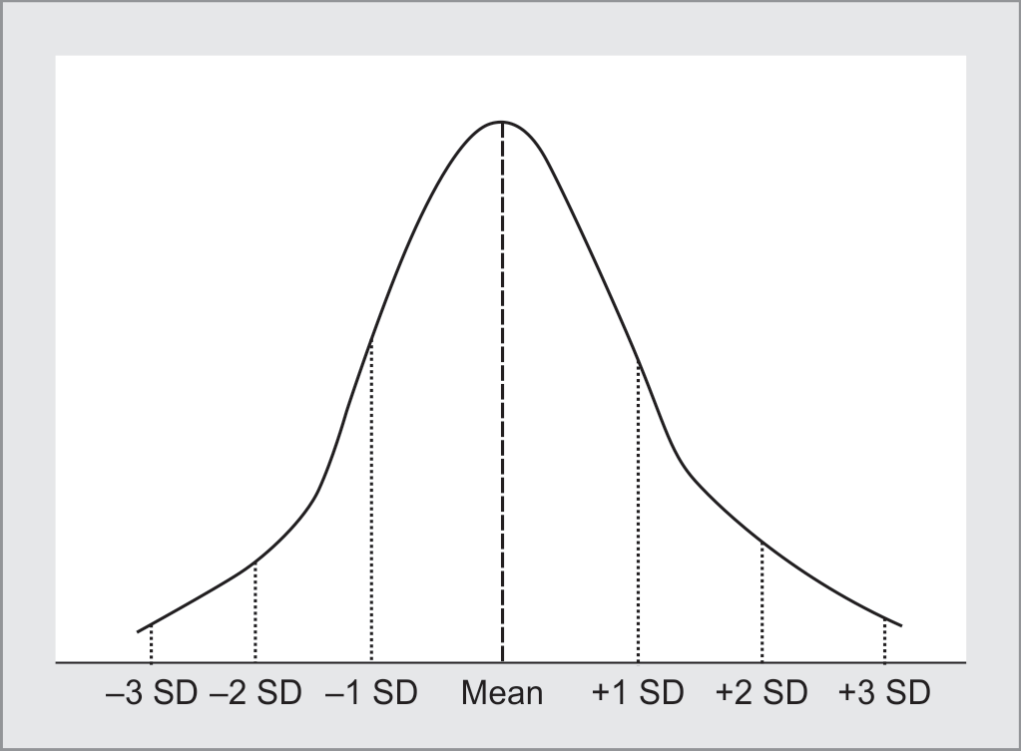
Normal distribution of data - the mean, median and mode are fairly close to each other

*Normally distributed data* is data that follows a symmetric bell-shaped curve (Gaussian distribution) (Fig. 1). Here, most of the data observations are centered around the middle and the curve gradually tapers on either side. Statisticians have formal methods of assessing normality of distribution. The simplest of these is to plot a histogram and visually inspect the distribution of the data. Other methods involve testing normality using a Q-Q plot or using the Kolmogorov Smirnov or the Shapiro Wilk tests.

**Fig. 2:**
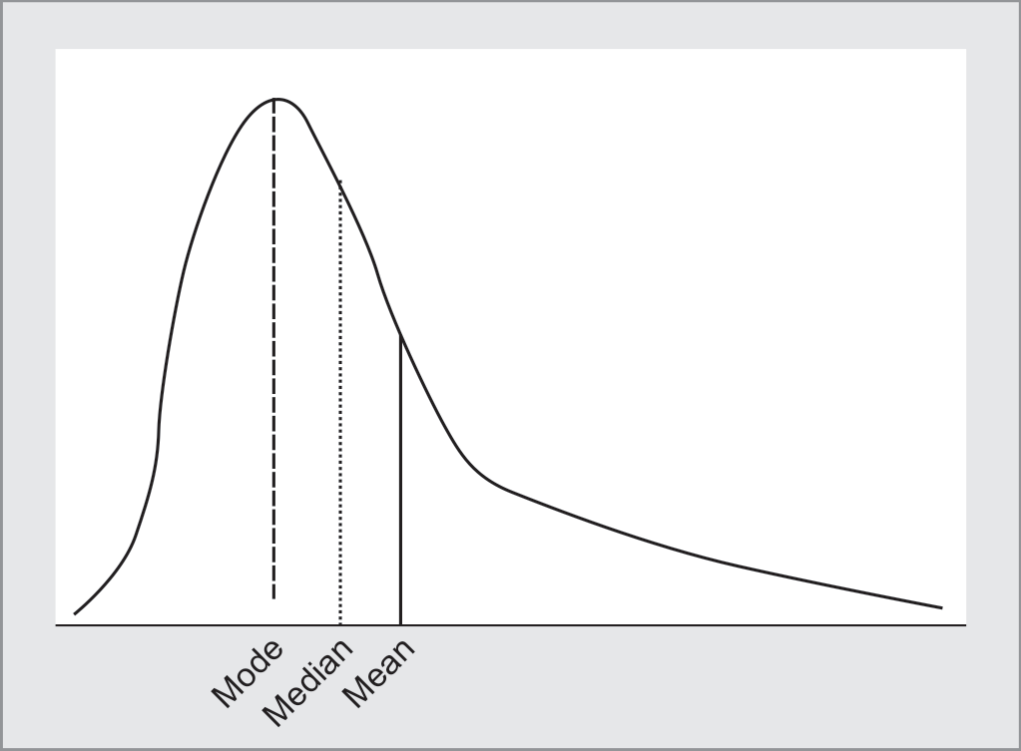
Skewed distribution of data - the mean, median and mode differ from each other

As against this, *skewed data* does not have a symmetric distribution and may have a long tail on one side (Fig. 2). Duration of surgery, duration of hospital stay or ICU stay are examples of data that is likely to be skewed.

*Note:* Distribution of data is important in the choice of statistical test for analysis. Tests that assume a normal or Gaussian distribution are known as parametric tests and tests that do not make assumptions about distribution are known as non-parametric or distribution-free tests. The former are in general more powerful tests.

### Summarizing Data

It is often difficult to represent individual data from multiple participants in a study unless these numbers are really small. For example, if there are 100 participants in a study, we cannot enumerate the ages of each of the participants. We therefore use certain measures to summarize this data and allow us to represent it easily.

For categorical data, we use percentages and depict this using bar graphs or pie charts. Refer to Table 1 of the article by Nielsen and colleagues, in which baseline characteristics of participants are listed.^2^ Gender, medical history and some of the characteristics of the cardiac arrest are examples of categorical data. These are shown as actual numbers with percentages in brackets.

For numerical data, we depict the ‘average’, which is “middle” of the data and the ‘dispersion’, which tells us how far away from the ‘average’ most of the values lie. If data is normally distributed, the average is the **arithmetic mean**. The spread of the data around the mean is depicted by the **standard deviation**. The larger the standard deviation, the more dispersed is the data (i.e., higher is the variance within the data). For example, in Table 1 of the article by Nielsen, age, body temperature, serum pH and serum lactate are all represented by the mean and standard deviation.^2^ Similarly, in Table 1 of the article by Nishikimi, several baseline characteristics are represented by their mean and standard deviation.^3^ For normally distributed data, the mean, median and mode are fairly close to each other.

For skewed data, the arithmetical mean does not give an idea of the true average. We therefore use another summary statistic called the **median** (or the 50th centile). For this, the data is arranged in ascending or descending order and divided into 100 equal parts (percentiles). The point that corresponds to the 50th percentile is the median (half of the observations are greater than and half are lesser than this value). The 25th to 75th percentiles give the **inter-quartile range**. In Table 1 of the article by Nielsen, time from cardiac arrest to event and Glasgow Coma scale are both examples of data that is represented by median and interquartile range.^2^ Graphically, a box-and-whisker plot or a stem-and-leaf plot help good visualization of skewed data. Readers can refer to Table 2A of the article by Nishikimi, in which duration of ICU stay is depicted by a box and whiskers plot.^3^ Unlike the mean, the median is not affected by extreme data points.

Rarely, one will find reference to another measure called the **mode**. The mode represents the observation that has the highest frequency. For example, if one was looking at age-wise distribution of incidence of a particular cancer, then the mode would tell us which age group had the highest incidence.

### Care with Data

The science of statistics begins with collection of data. Apart from understanding data types, distributions and how to summarize data, we need to be meticulous in the process of collection and extremely careful in writing, entering data into software or excel sheets or transcribing data, as errors can seriously impact conclusions and the consequent decision making in patient care.
